# *M.globosa* promotes lung cancer progression and M2 macrophage polarization through oxidative phosphorylation

**DOI:** 10.1038/s41698-026-01528-5

**Published:** 2026-06-04

**Authors:** Junqi Yi, Yiming Zhao, Zhengjiang Li, Anqi Chen, Ziying Tang, Leliang Zheng, Huabo Ge, Qian Yu, Wenliang Liu, Juanjuan Xiang, Jingqun Tang

**Affiliations:** 1 Department of thoracic surgery, the Second Xiangya Hospital, Central South University, Changsha, Hunan China; 2 NHC Key Laboratory of Carcinogenesis and the Key Laboratory of Carcinogenesis and Cancer Invasion of the Chinese Ministry of Education, Cancer Research Institute, School of Basic Medical Science, Central South University, Changsha, Hunan China; 3 Hunan Key Laboratory of Early Diagnosis and Precise Treatment of Lung Cancer, Changsha, Hunan China

**Keywords:** Cancer, Diseases, Microbiology

## Abstract

The lungs are colonized by a variety of microbes which play a significant role in lung cancer progression. In this study, we conducted an in-depth analysis of metagenomic sequencing data obtained from alveolar lavage fluid (ALF) samples of patients with non-small-cell lung cancer (NSCLC) at different clinical stages. The nested qPCR was used to validate the abundance of key fungi and establish a correlation between fungi abundance and patient prognosis. We found that elevated levels of *M.globosa* correlated with patients at stage1B-3 and worse prognosis. *M.globosa* enhanced the proliferation of lung cancer cells and promoted tumor growth in vivo by promoting M2-like macrophage polarization, which was primarily driven by oxidative phosphorylation (OXPHOS) activation. The inhibition of OXPHOS in tumor-bearing mice using metformin significantly retarded the tumor growth induced by *M. globosa*. Together, our clinical observations and experimental findings suggest that intracellular *M. globosa* infection may contribute to lung cancer progression through immunometabolic remodeling of macrophages.

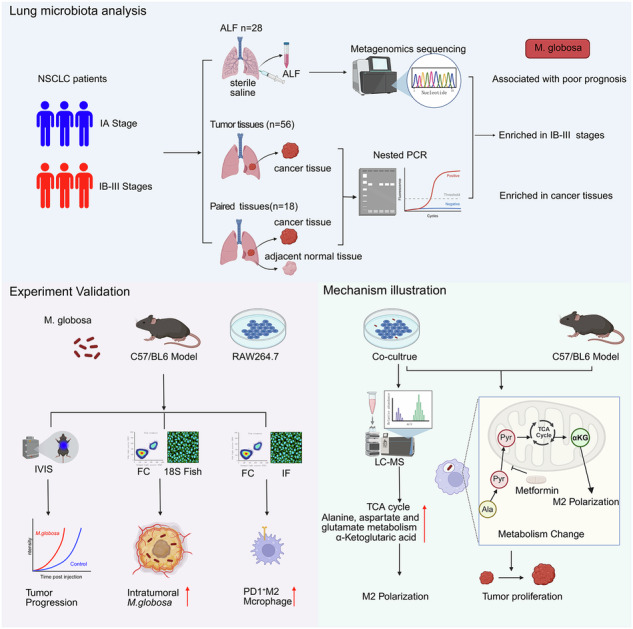

## Introduction

It is now well established that the lungs are not sterile, rather, they are colonized by a variety of microbes including bacteria, archaea, viruses and fungi. The assemblage of living microorganisms present in a defined environment is referred to as “microbiota” and microbiome refers to the collective genomes of the microorganisms in a particular environment^1,2^. The lung microbiome participates in immune responses and maintains homeostasis. Lung cancer is responsible for 18.7% of all cancer deaths, more than any other malignancy^3^. The associations between microbes and tumors have become increasingly evident. A direct link between infection and the development of neoplasia has been demonstrated, for example, EB virus and lymphoma^4^. Our previous study has performed metagenomic sequencing and found the association between specific microbes and lung cancer^5^. The mycobiota represents the important group of eukaryotes in the human microbiota. Pan-cancer analysis of multiple body sites revealed tumor-associated mycobiome at up to 1 fungal cell per 10^4^ tumor cells^6^. Fungi are detected in 35 cancer phenotypes and are often intracellular^7^. Ascomycota and Basidiomycota phyla dominate the cancer intratumoral mycobiome^7^. Published data have shown that the mycobiome plays a significant role in the development of cancers^6–10^. The microbiome dysbiosis is associated with lung cancer^11,12^. In lung cancer, Blastomyces was associated with tumor tissues^13^. However, the roles of mycobiota and specific fungi in lung cancer remain to be elucidated.

*Malassezia globosa* (*M.globosa*) is a kind of basidiomyces yeast species. It was thought that *M.globosa* is specialized to live on skin and associated with skin diseases^14^. Recent studies found that the dysbiosis in fungal communities is always characteristic of high level of *Malassezia* in inflammatory diseases such as IBD and various cancers^15^. It has been demonstrated that *M.globosa* is enriched in several cancers including pancreatic duct adenocarcinoma, colorectal cancer, head and neck squamous cell carcinoma^16^. Our previous research found the roles of *M.globosa* as the core nodule in mycobiota of non-small-cell lung cancer (NSCLC) patients^17^. The crucial link between *M.globosa* and lung cancer remains unclear.

In this study, we found that the abundance of *M.globosa* was associated with lung cancer progression. The intracellular infection of *M. globosa* polarized macrophages toward an M2-like phenotype through activation of oxidative phosphorylation (OXPHOS). Inhibition of OXPHOS with metformin could reverse *M. globosa*-induced M2-like polarization and significantly retard tumor growth, both in vitro and in vivo.

## Results

### The lung microbiota displays different composition among lung cancer patients with different clinical staging

**Fig. 1:**
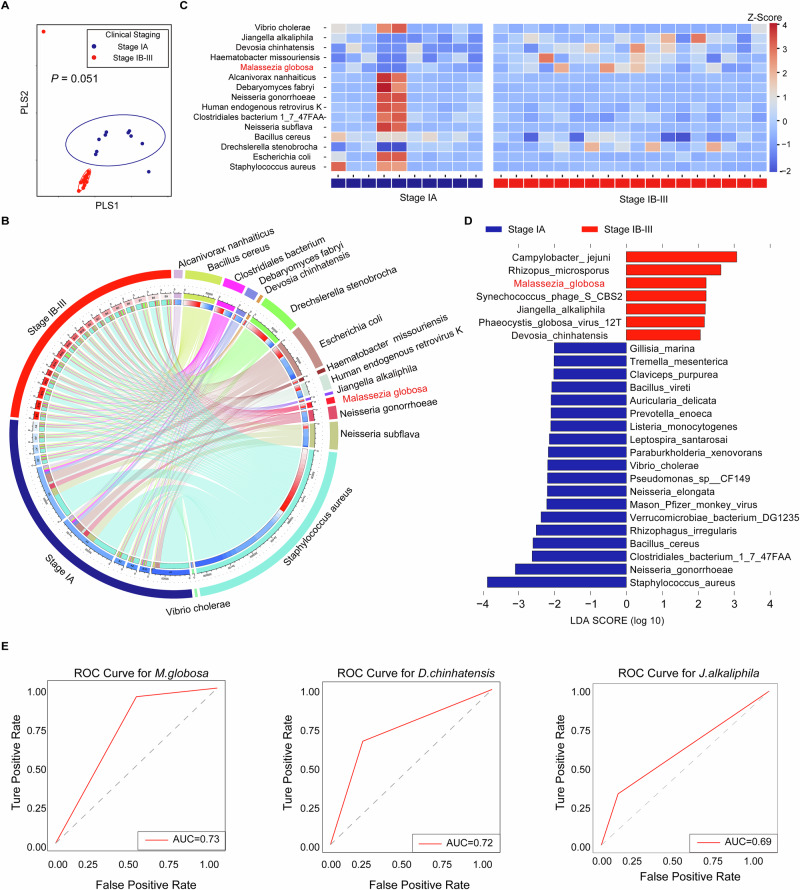
The lung microbiota displays different composition among lung cancer patients with different clinical staging. The shotgun metagenomical data was analyzed in 28 lung cancer patients with different clinical stages. **A** The Bray-Curtis dissimilarity analysis in lung microbiome in NSCLC patients; **B** The heatmap was built from the top 15 microbe species with the most fold changes in relative abundance; **C** The differential species were identified by DESeq2 (|log2FC | > 1 and adj. *p* < 0.05); **D** LEfSe analysis of lung microbiota in NSCLC patients with stage1A and stage1B-3. The histogram represents significantly different taxonomic units between patients with stage1A (blue) and stage1B-3 (red). Taxa with LDA scores > 2 are shown; **E** ROC curves of *M.globosa, D.chinhatensis and J.alkaliphila* for predicting clinical stages in NSCLC patients.

In order to examine the relationship between lung microbiota and lung cancer progression, we analyzed shotgun metagenomic data in 28 lung cancer patients with different clinical stages (SRA, PRJNA714488). We collected the lavage fluid from alveoli of patients undergoing lobectomy. The 28 patients were divided into stage 1 A and other stages ranging from 1B to stage 3(Supplementary Fig. 1A, 1B). As depicted in Supplementary Fig. 1C, the lung microbiota comprised Bacteria (47.01%), Eukaryota(1.12%), Viruses (0.66%), Archaea (0.20%) and unclassified (51.02%). Among all taxa, the most dominant phyla across all samples were Firmicutes, Proteobacteria and Actinobacteria (Supplementary Fig. 1D). We compared the diversity of the lung microbiome across different clinical stages. The diversity indices including species richness, Shannon-Wiener index, Simpson dominance index and Pielou evenness index showed a decreasing trend in stage1B-3 patients (Supplementary Fig. 1E). However, the diversity was not statistically significant between stage 1 A and stage 1B-3 patients, likely due to the limited sample size. The Bray-Curtis dissimilarity analysis appeared to separate samples into 2 groups based on species abundance (Fig. 1A). The differential species with the most fold-changes in relative abundance were identified using DESeq2^18^, including 6 species enriched in stage 1B-3 patients and 7 species enriched in stage 1 A patients (Fig. 1B and 1C). Species that differed significantly between stage 1 A and other stages of lung cancer patients were presented in Supplementary Fig. 1F, as identified by the Wilcoxon test. We also performed LEfSe analysis which demonstrated 7 differential species enriched in stage 1B-3 patients including *M.globosa*, *D.chinhatensis*, and *J.alkaliphila*, which were also found in DESeq2 analysis(Fig. 1D). To further assess the diagnostic potential of these species, receiver operating characteristic (ROC) analysis was carried out. The area under the ROC curve (AUC) for *M. globosa*, *D. chinhatensis*, and *J. alkaliphila* was 0.73, 0.72, and 0.69, respectively. As shown in Fig. 1F, the abundance of *M. globosa* was correlated with clinical cancer stages. Fisher’s exact test further validated that high *M. globosa* abundance was significantly associated with clinical cancer stages (*P* = 0.0183), whereas *D. chinhatensis* and *J. alkaliphila* showed no such association (Supplementary Table 1). To examine potential confounding, we assessed the association between *M. globosa* abundance and key clinical characteristics. As summarized in Supplementary Table 2, no significant associations were found with gender, age, smoking history and histological subtype. Collectively, these findings imply that certain lung microbiota species, especially *M. globosa*, might be linked to the progression of lung cancer.

### M.globosa is enriched in stage 1B-3 patients with NSCLC

**Fig. 2:**
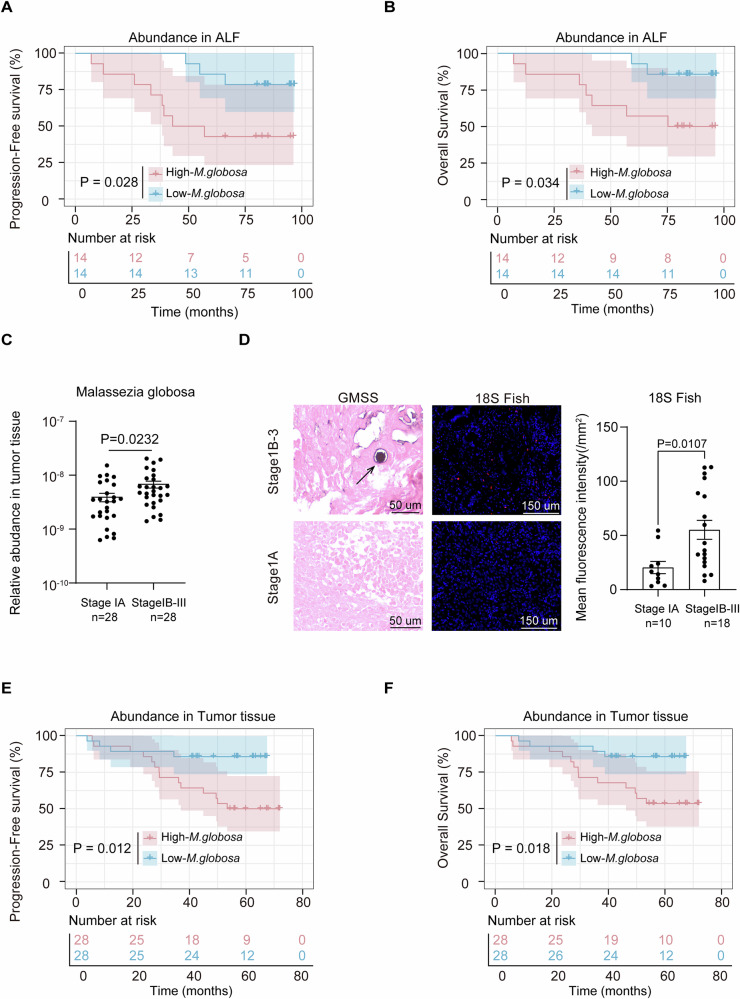
*M.globosa* is enriched in stage1B-3 patients with NSCLC. Progression-free survival (**A**) and Overall Survival (**B**) of NSCLC patients stratified into *M. globosa*-high or -low based on median ALF abundance, *n* = 28. **C** The abundance of *M. globosa* in tumor tissues from an independent cohort using qPCR, *n* = 56. **D** Fluorescence in situ hybridization for 18S rRNA and Grocott methenamine silver staining in tumor tissues from NSCLC patients (ALF, *n* = 28). Representative images showing fungal localization in the cancer tissues. The histogram represents significantly different fungal abundance in stage 1A and stage 1B-3 patients. *n* = 28; Progression-free survival (**E**) and Overall survival (**F**) of NSCLC patients stratified into *M. globosa*-high or -low based on median tumor tissue abundance, *n* = 56. Data represent mean ± S.E.M and statistics used unpaired two-tailed *t* test.

We then divided the above 28 patients into two groups (low-abundance and high-abundance) based on the median abundance of the three species in alveoli lavage fluid (ALF). Higher abundance of *M.globosa* was correlated with poorer progression-free survival (*P* = 0.028) and shorter overall survival in NSCLC patients (*P* = 0.034), whereas no such correlation was observed for *D.chinhatensis and J.alkaliphila* (Fig. 2A, B, Supplementary Fig. 2A, 2B). Multivariate Cox regression revealed that *M. globosa* abundance was an independent prognostic factor for patient survival(PFS: HR = 3.945, 95% CI:1.104 to 18.42, *P* = 0.0474) (Table 1). To investigate whether *M. globosa* is present in lung cancer tissue, we performed specific nested PCR in two independent cohorts: one consisting of 18 patients with paired tumor and adjacent normal tissues, and another of 56 patients with different clinical stages. The amplification curves and melting curves verified the specificity of the primers (Supplementary Fig. 2C, 2D). Compared with matched adjacent normal tissues, cancer tissues displayed a higher abundance of *M. globosa*, suggesting enrichment of this fungus in tumor tissues (*P* = 0.0139, *n* = 18; Supplementary Fig. 2E). As shown in Fig. 2C, the higher abundance of *M.globosa* was observed in stage1B-3 patients compared to stage 1A patients (*P* = 0.0232, *n* = 56). Supplementary Table 3 shows no significant association between *M. globosa* abundance and gender, age, smoking history, or histological subtype. Fluorescence in situ hybridization (FISH) for 18S rRNA (a fungal biomarker) and Grocott methenamine silver staining (GMSS) revealed a greater abundance of colonized fungi in tumor tissue from stage 1B-3 patients than from stage 1 A patients (*P* = 0.0107, *n* = 28). This spatial distribution further supported the potential association between fungi abundance and lung cancer progression (Fig. 2D). The higher abundance of *M. globosa* in cancer tissues was correlated with poorer progression-free survival (*P* = 0.012) and shorter overall survival in NSCLC patients (*P* = 0.018) (Fig. 2E, F). Univariate Cox regression analysis identified *M. globosa* abundance as a significant predictor of poor progression-free survival (PFS) (HR = 3.752, 95% CI: 1.345–13.24, *P* = 0.0198). However, Multivariate Cox regression did not reach statistical significance when adjusting for factors including TNM staging and tumor size (HR = 2.490, 95% CI: 0.791–9.616, *P* = 0.1444)(Supplementary Table 4). These results suggest that *M. globosa* present in cancer tissue is the factor associated with the prognosis of NSCLC patients. Taken together, the enrichment of *M. globosa* in stage 1B-3 patients and its correlation with poor prognosis underscore its potential association with lung cancer progression.

**Table 1:** Prognostic factors in NSCLC patients (*n* = 28)

Variable	PFS						OS					
	Univariate analysis			Multivariate analysis			Univariate analysis			Multivariate analysis		
	HR	95% CI	*P*	HR	95% CI	*P*	HR	95% CI	*P*	HR	95% CI	*P*
**Gender**												
Male/Female	0.5877	0.1691 to 1.955	0.3808				0.3645	0.07686 to 1.383	0.1538			
**Age**												
≥60/ <60	1.299	0.3908 to 4.511	0.6663				2.404	0.6309 to 11.44	0.2165			
**Somking history**												
Ever/Never	2.175	0.6252 to 7.243	0.2005				3.06	0.8082 to 12.39	0.096			
**Tumor Size**												
＞ 3 cm/≤3 cm	3.046	0.879 to 10.18	0.0676				2.641	0.6506 to 10.04	0.1495			
**TNM Stages**												
＞ T1/T1	5.023	1.288 to 33.04	**0.0395**	3.968	0.9904 to 26.46	0.0829	8.482	1.549 to 157.6	**0.0442**	6.1	1.073 to 115.0	0.0931
***M.globosa*** **Abundance**												
High/Low	4.887	1.399 to 22.47	**0.0200**	3.945	1.104 to 18.42	**0.0474**	5.654	1.358 to 38.08	**0.0313**	3.959	0.9287 to 27.11	0.0919
***J.alkaliphila*** **Abundance**												
High/Low	3.71	1.067 to 17.00	0.0537				2.592	0.6807 to 12.33	0.1794			
***D. chinhatensis*** **Abundance**												
High/Low	0.3461	0.07556 to 1.202	0.1182				0.4815	0.1014 to 1.829	0.3021			
**Pathological type**												
Squamous cell carcinoma / Adenocarcinoma	2.04	0.3101 to 7.961	0.3631				2.677	0.3972 to 11.16	0.2212			

The bold values indicate *P* values that are less than 0.05, which were considered statistically significant. Specifically, the bold *P* values are: 0.0395, 0.0200, 0.0474, 0.0442, and 0.0313.

### Lung colonization of M.globosa promotes lung cancer progression

**Fig. 3:**
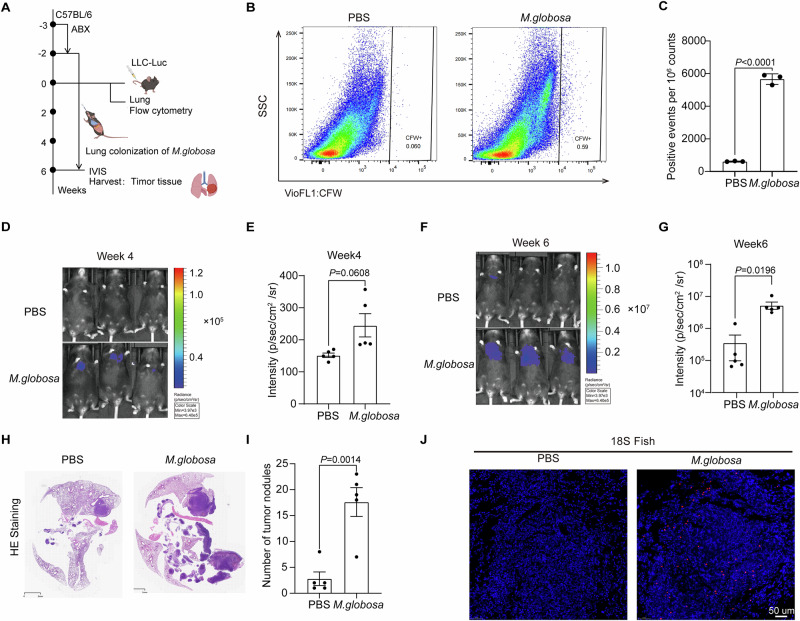
Lung colonization of *M.globosa* promotes lung cancer progression. **A** Schematics of the *M.globosa* transplantation experiments in C57BL/6 LLC mice. **B** Flow cytometry analysis of lung colonization of CFW-labeled *M.globosa*. **C** The number of CFW positive cells was shown in histogram. **D** Bioluminescence images of tumor-bearing mice at 4th week. **E** The fluorescence intensity of live imaging at 4th week. **F** Bioluminescence images of tumor-bearing mice at 6th week. **G** The fluorescence intensity of live imaging at 6th week. **H** Representative images of H&E-stained lung sections. Scale bar:2000 μm. **I** The number of tumor nodules were calculated; *n* = 5. **J** Representative FISH staining for 18 s rRNA in lung tumor tissues. Scale bar: 50 μm Data represent mean ± S.E.M. Statistics used unpaired two-tailed Student’s *t*-test. A *P*-value less than 0.05 were considered to be significant.

We then used LLC murine lung carcinoma syngeneic model to evaluate the effects of *M.globosa* on cancer progression. The treatment protocol was shown in Fig. 3A. The mice were divided into two groups: the PBS control group and *M. globosa* group, which received intranasal instillation of calcofluor white (CFW)-labeled *M. globosa*. One week later, we used flow cytometry to measure the lung colonization of CFW-labeled *M.globosa*. As shown in Fig. 3B, C, CFW-positive cells were detected in the lungs of *M.globosa-*treated mice, suggesting the colonization of the lungs by *M.globosa* (*P* < 0.0001). We then intravenously injected luciferase-LLC cells into *M.globosa*-colonized C57BL/6 mice. At 4 weeks and 6 weeks post-injection, the in vivo imaging was used to measure the growth of cancer cells. Mice treated with *M. globosa* showed enhanced tumor growth compared with PBS-treated controls in a time-dependent manner (Fig. 3D–G). At week 6, bioluminescence intensity, a proxy for tumor growth, was significantly higher in *M. globosa*-treated mice than in PBS-treated controls (*P* = 0.0196, Fig. 3F, G). H&E staining revealed more tumor nodules in the *M. globosa*-treated group than in the PBS-treated group (Fig. 3H). The number of tumor nodules was calculated (Fig. 3I, *P* = 0.0014). The FISH for 18S rRNA revealed that the fungal organisms were detected in lung tumor tissues in *M.globos*a-treated group (Fig. 3J). Collectively, these results indicate that pulmonary colonization by *M. globosa* promotes the progression of LLC lung carcinoma in vivo.

### Intracellular infection of *M.globosa* induces oxidative phosphorylation of macrophages

**Fig. 4:**
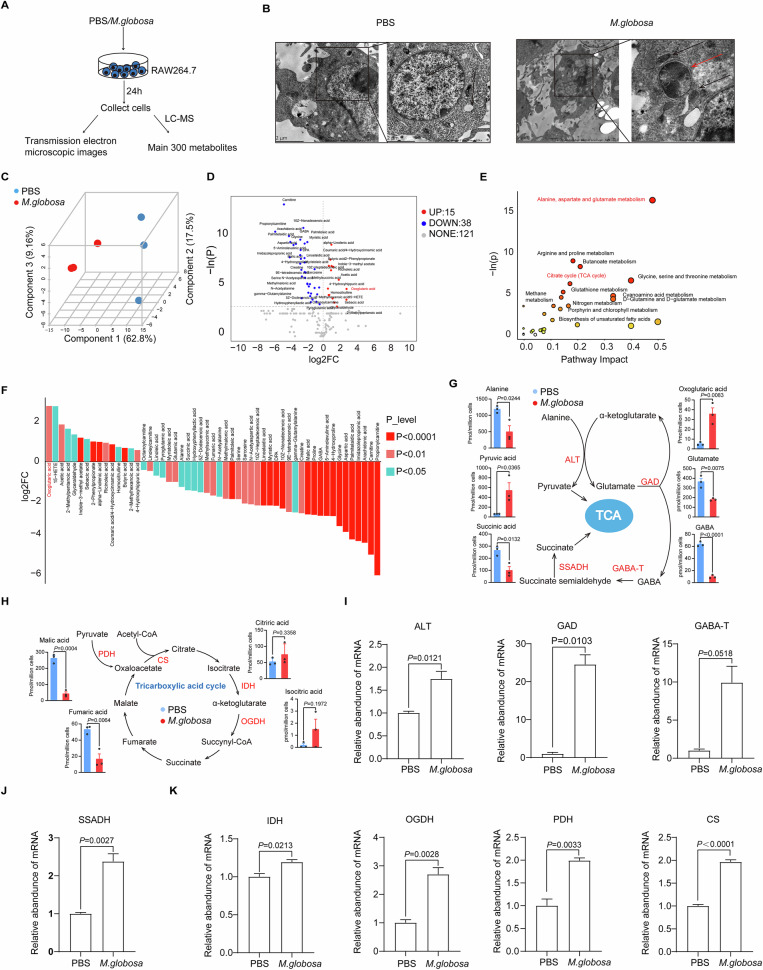
Intracellular infection of *M.globosa* induces oxidative phosphorylation of macrophages. The macrophage raw264.7 cells were infected with *M.globosa*. **A** Schematics of *M.globosa* infected with RAW264.7 experiments. **B** The representative electron microscopic images of *M.globosa*-infected macrophages. The black arrow indicated mitochondrial; The red arrows indicated *M.globosa*. **C** 3D PCA of metabolites in *M.globosa*-infected macrophages and uninfected control macrophages. **D** Volcano plot of significantly differentially expressed metabolites in *M.globosa*-infected macrophages and uninfected control macrophages; **E** The KEGG pathway analysis of metabolites with significant difference; **F** The significantly differentially expressed metabolites in *M.globosa*-infected macrophages and uninfected control macrophages; **G** The abundance of metabolites in Alanine, aspartate and glutamate metabolism. **H** The abundance of metabolites in TCA cycle. **I** The expression of key enzymes (ALT, GAD, and GABA-T) involved in alanine, aspartate, and glutamate metabolism at the mRNA level in RAW264.7 cells treated with *M. globosa* or PBS. **J** The expression of SSADH involved in alanine, aspartate, and glutamate metabolism at the mRNA level in RAW264.7 cells treated with *M. globosa* or PBS. **K** The expression of key enzymes (OGDH, IDH, PDH and CS) involved in the TCA cycle at the mRNA level in RAW264.7 cells treated with *M. globosa* or PBS. Data are presented as mean ± S.E.M. (*n* = 3). Statistics used unpaired two-tailed Student’s t-test.

Macrophages are the first line of defense against microbes^19^. We then infected RAW264.7 macrophages in vitro with *M.globosa* (Fig. 4A). Optical microscopic images, fluorescence microscope images and transmission electron microscopic images all indicated the intracellular infection of *M.globosa* (Fig. 4B, Supplementary Fig. 3A and 3B) .The intracellular infection of *M. globosa* led to mitochondrial swelling in macrophages (Fig. 4B), indicating potential metabolic stress or activation.

We then examined the metabolic alterations of macrophages after infection of *M. globosa*. About 24 hours after infection of *M.globosa*, macrophages were harvested and analyzed by LC-MS-based metabolomics. The main 300 metabolites in the infected macrophages were analyzed. The most altered metabolites included amino acids, carbohydrates, fatty acids, SCFAs, etc. (Supplementary Fig. 3C and 3D). The 3D OPLS-DA plots obtained from untargeted metabolomics presented a separation between infected and uninfected macrophages (Fig. 4C). We then performed Mann–Whitney *U* test to obtain the significantly different metabolites between infected and uninfected macrophages. A *P*-value less than 0.05 and an absolute log2 fold change bigger than 0 were considered to be significant. As shown in Fig. 4D, a total of 53 metabolites were changed significantly between these two groups, 15 of which were significantly increased and 38 of which were significantly decreased in infected macrophages compared to those in uninfected macrophages.

Metabolic pathway enrichment analysis revealed that the influenced metabolism pathway mainly enriched in Alanine, aspartate and glutamate metabolism, Glycine, serine and threonine metabolism, TCA cycle et al. (Fig. 4E). The differentially expressed metabolites, including Oxoglutaric acid, Glyceraldehyde, and alpha-Linolenic acid, were shown in Fig. 4F and Supplementary Fig. 3E. Enzymes involved in oxidative phosphorylation, including pyruvate carboxylase, citrate synthase, succinate exchange, pyruvate kinase, were among the highly enriched pathway upon *M.globosa* infection (Supplementary Fig. 3F). The differentially expressed metabolites with most fold change value including Alanine(*P* = 0.0244), Pyruvate(*P* = 0.0365), Succinate(*P* = 0.0132), α-ketoglutarate (*P* = 0.0083), Glutamate(*P* = 0.0075), GABA(*P* < 0.0001), Citrate(*P* = 0.0083), Malate(*P* = 0.0004), Fumarate(*P* = 0.0064), among others, were shown in Fig. 4G, H. Thus, infection with *M. globosa* triggers significant metabolic reprogramming in macrophages, characterized by activation of the TCA cycle.

To verify these metabolic shifts, the qPCR assay was used to measure the levels of key enzymes involved in TCA cycle and Alanine, aspartate and glutamate metabolism. As shown in Fig. 4I, J, and K, ALT(*P* = 0.0121), GAD(*P* = 0.0103), SSADH(*P* = 0.0027), OGDH(*P* = 0.0028), IDH(*P* = 0.0213), PDH(*P* = 0.0033), and CS(*P* *<* 0.0001) were significantly upregulated following infection with M. globosa.

### Infection of *M.globosa* induces macrophage M2-like polarization in vitro and in vivo

**Fig. 5:**
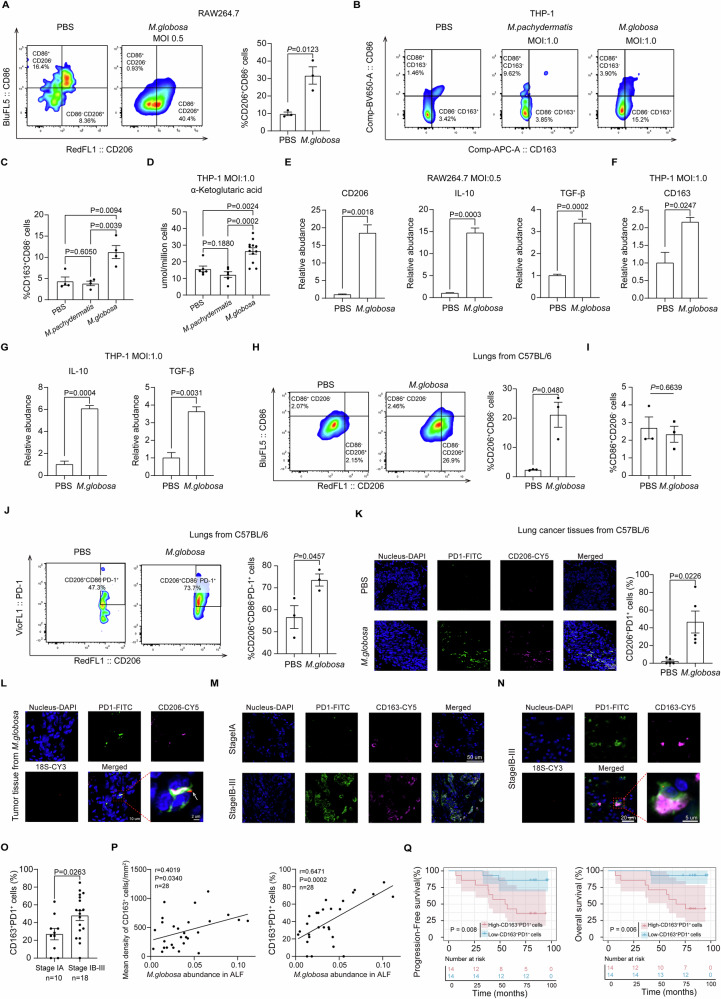
Infection of *M.globosa* induces M2-like polarization of macrophages. **A** Flow cytometry analysis of CD86^+^ vs CD206^+^ cells macrophages in RAW264.7 cells after *M.globosa* infection, and statistical analysis of the percentage of M2 macrophages in *M.globosa-*infected RAW264.7 cells; *n* = 3. MOI: multiplicity of infection, (**B**) Flow cytometry analysis of CD86^+^ vs CD163^+^ macrophages in THP-1-derived macrophages treated with *M. globosa*, PBS or *M. pachydermatis*. **C** Statistical analysis of the percentage of M2-like macrophages was shown on the right;, *n* = 4; **D** Intracellular levels of α-ketoglutaric acid in THP-1-derived macrophages treated with *M. globosa*, PBS or *Malassezia pachydermatis*, *n* = 12; **E** qPCR analysis of CD206, IL-10, and TGF-β expression in RAW264.7 cells, *n* = 3. **F**–**G** qPCR analysis of CD163, IL-10, and TGF-β expression in THP-1-derived macrophages at 24 h post-infection, *n* = 3. **H** Flow cytometry analysis of macrophages from the lungs of C57BL/6 mice; and statistical analysis of the percentage of M2-like macrophages was shown on the right. **I** Statistical analysis of the percentage of M1-like and M2-like macrophages from the lungs of C57BL/6 mice; **J** Flow cytometry analysis of CD206^+^ PD-1^+^ macrophages and statistical analysis of the percentage of PD-1^+^ M2-like macrophages from the lungs of C57BL/6 mice. All data are presented as mean ± SEM (*n* = 3). **K** Immunofluorescence staining for PD-1 and CD206 in lung cancer tissues of *M.globosa*-treated LLC syngeneic C57BL/6 mice(*n* = 5). **L** Triple staining for 18S rRNA and immune markers (CD206, PD1) in lung cancer tissues of *M.globosa*-treated LLC syngeneic C57BL/6 mice(*n* = 5). **M** Immunofluorescence staining for PD-1 and CD163 in cancer tissues from different clinical stages. **N** The localization of fungi within CD163^+^PD-1^+^ macrophages in cancer tissues from NSCLC patients were detected by immunofluorescence. **O** Statistical analysis of the percentage of CD163^+^PD-1^+^ macrophages in cancer tissues from different clinical stages were shown on the right (*n* = 28). **P** Correlation of the relative abundance of *M.globosa* in ALF and the expression of CD163^+^ macrophages and CD163^+^PD-1^+^ macrophages in lung cancer tissues of NSCLC patients (*n* = 28). **Q** Kaplan–Meier survival analysis of PFS and OS in NSCLC patients stratified by the proportion of CD163^+^PD-1^+^ macrophages (*n* = 28). Data represent mean ± S.E.M. Statistics used unpaired two-tailed t test .

The activation of oxidative phosphorylation implied a shift toward the M2-like polarization of macrophages^20^. In vitro infection of RAW264.7 macrophages and human THP-1-derived macrophages caused a significant increase in CD86^-^CD206^+^ M2-like (mouse; *P* = 0.0123) and CD86^-^CD163^+^ M2-like (human) macrophages (Fig. 5A-C). The infection of THP-1-derived macrophages by *M. globosa* led to an increase in M2-like macrophages (vs. PBS:*P* = 0.0094; vs. *M. pachydermatis:* *P* = 0.0039) and the intracellular levels of α-ketoglutaric acid (vs. PBS:*P* = 0.0024; vs. *M. pachydermatis*: *P* = 0.0002, Fig. 5D); however, *M. pachydermatis* did not induce M2-like polarization and the associated metabolic alterations. These findings indicate the unique role of *M. globosa* in macrophage polarization rather than a general effect from any fungi. Consistently, qPCR analysis verified markedly increased expression of M2-related markers after *M. globosa* infection, such as CD206 or CD163, IL-10, and TGF-β in both mouse and human macrophages (Fig. 5E–G).

We then assessed the polarization of macrophages in the lungs of *M.globosa*-treated mice. The mice were treated with *M. globosa*, and lung tissues were subjected to flow cytometry. As shown in Fig. 5H, I, the lungs of *M. globosa-treated* mice displayed increased infiltration of CD45^+^/CD11B^+^/ CD86^-^/CD206^+^ cells compared to control PBS mice, suggesting M2-like polarization induced by *M. globosa* (*P* = 0.048). Conversely, the proportion of M1-like phenotype macrophages in the lungs showed no significant difference between *M. globosa-treated* and control mice (Fig. 5I). We further examined the expression of PD-1 on these macrophages, given its reported role in suppressing antitumor immunity^21,22^. We found that PD-1 positive M2-like macrophages were significantly increased in the lungs of *M.globosa* infected mice (*P* = 0.0457; Fig. 5J). In *M.globosa*-treated LLC lung cancer tissues, immunofluorescence staining (CD206, PD1) revealed that *M. globosa* treatment increased CD206^+^/PD-1^+^macrophages (Fig. 5K, *P* = 0.0226). Triple staining for 18S rRNA and immune markers also suggested that fungi were predominantly localized within CD206^+^/PD-1^+^macrophages (Fig. 5L). Clinically, lung cancer tissues from NSCLC patients with clinical stage 1B-3 showed higher infiltration of CD163^+^/PD-1^+^ macrophages (Fig. 5M,O, *P* = 0.0263). In cancer tissues from patients with stage 1B-3, the localization of fungi within CD163^+^/PD-1^+^ macrophages was also detected (Fig. 5N). Correlation analysis revealed that the abundance of *M. globosa* in ALF was positively correlated with the infiltration of CD163^+^ macrophages (*r* = 0.4019, *P* = 0.034) and, more strongly, with CD163^+^/PD-1^+^ macrophages (Fig. 5P, *r* = 0.6471, *P* = 0.0002). Poorer progression-free survival (*P* = 0.008) and overall survival (*P* = 0.006) in lung cancer patients were correlated with a higher proportion of CD163^+^/PD-1^+^ macrophages (Fig. 5Q). These findings suggest that *M. globosa* infection induces M2-like polarization of macrophages accompanied by elevated PD-1 expression in experimental models. Increased infiltration of PD-1^+^ macrophages correlates with poor prognosis in lung cancer patients, implying a link between *M. globosa*-driven immune modulation and lung cancer progression.

### Metformin suppresses M2-like macrophage polarization and lung cancer progression induced by *M.globosa* through inhibition of oxidative phosphorylation

**Fig. 6:**
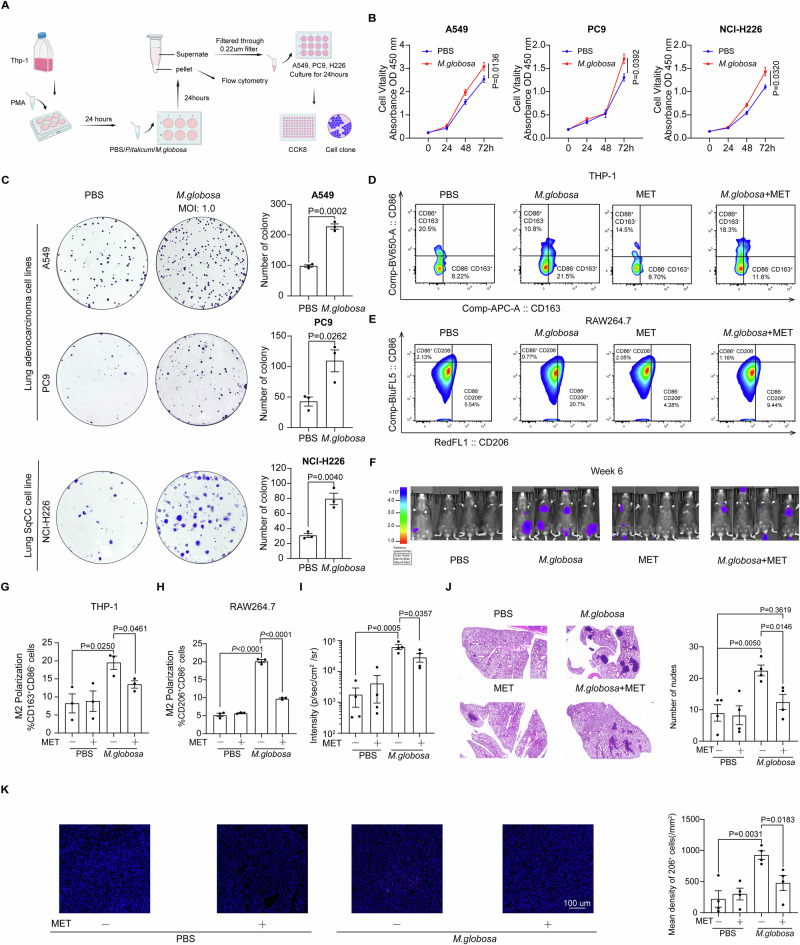
M2-like polarization of macrophage induced by M.globosa is oxidative phosphorylation dependent. **A** Schematic illustration of the workflow. Human THP-1-derived macrophages were infected with *M. globosa*, and the resulting macrophage-conditioned medium was used to treat lung cancer cells. **B** CCK-8 analysis of cell viability in A549, PC9, and NCI-H226 cells treated with conditioned medium from *M.globosa*-infected THP-1-derived macrophages (*n* = 3). **C** Colony formation assay showing the clonogenic potential of A549, PC9, and NCI-H226 cells treated with conditioned medium from *M. globosa*-infected THP-1-derived macrophages.SqCC: Squamous Cell Carcinoma, *n* = 3. **D** Flow cytometry analysis of CD86^+^ vs CD163^+^ cells in THP-1-derived macrophages treated with metformin. **E** Flow cytometry analysis of CD86^+^ vs CD206^+^ cells in RAW264.7 macrophages treated with metformin. **F** Representative bioluminescence images of tumor-bearing mice at week 6. Percentage of M2-like macrophages in *M. globosa*-infected THP-1 (**G**) and RAW264.7 (**H**), *n* = 3. macrophages treated with or without metformin. **I** The fluorescence intensity of live imaging at 6th week. **J** Representative images of H&E-stained lung sections and the number of tumor nodules were calculated;Scale bar:200 μm. **K** Representative images of IF staining and mean density of CD206 in lungs, Scale bar:50μm; *n* = 4.Data represent mean ± S.E.M and statistics used unpaired two-tailed t test .

We subsequently explored the in vitro impact of *M.globosa-*infected macrophages on cancer cell proliferation. Human THP-1-derived macrophages were infected with *M.globosa* and the resulting macrophage-conditioned medium was used to treat lung cancer cells (Fig. 6A). CCK-8 assays revealed that conditioned medium from *M. globosa-*infected macrophages enhanced the viability of lung cancer cells (A549: *P* = 0.0136; PC9: *P* = 0.0392; NCI-H226: *P* = 0.0320) (Fig. 6B). Colony formation assays demonstrated that *M. globosa* infection triggered the clonogenicity of lung cancer cells (A549: *P* = 0.0002; PC9: *P* = 0.0262; NCI-H226: *P* = 0.0040) (Fig. 6C).

To determine whether the pro-tumor effect of *M. globosa* is mediated by metabolic reprogramming of macrophages toward M2-like polarization, we used metformin, an oxidative phosphorylation inhibitor, to interfere with the metabolic changes induced by *M.globosa*. THP-1 or RAW264.7 macrophages infected with *M.globosa* were treated with metformin (Fig. 6D, 6E). Remarkably, metformin treatment led to a notable decrease in the proportion of M2-like phenotype macrophages after *M.globosa* infection for 48 hours(THP-1:*P* = 0.0461; RAW264.7:*P* < 0.0001)(Fig. 6G, H). The reversal effect of metformin on the enhanced expression of CD206 (*P* = 0.0088) and TGF-β (*P* = 0.0187)induced by *M.globosa* was validated by qPCR (Supplementary Fig. 4A). The addition of metformin to *M.globosa*-infected macrophages significantly inhibited the enzymes involved in oxidative phosphorylation, including, OGDH(*P* = 0.0002), GAD(*P* = 0.0004), GABA-T(*P* = 0.0039) and ALT(*P* < 0.0001) (Supplementary Fig. 4B). These compelling findings underscore metformin’s potential to suppress *M.globosa*-induced oxidative phosphorylation activation and subsequently inhibit macrophage M2-like polarization. Importantly, metformin treatment decreased the clonogenicity of lung cancer cells induced by *M.globosa* infection(A549: *P* = 0.0105; PC9: *P* = 0.0041; NCI-H226: *P* = 0.0345) (Supplementary Fig. 4C). To provide a more comprehensive understanding, we conducted in vivo bioluminescence assays to assess the impact of metformin on *M.globosa*-induced cancer progression. We utilized a murine lung carcinoma syngeneic model injected with luciferase-LLC cells. Subsequent treatment of the mice with *M.globosa*, followed by metformin administration, allowed for the monitoring of cancer progression. The results revealed a significant inhibition of cancer growth in metformin-treated mice(*P* = 0.0357), as evidenced by a substantial reduction in bioluminescence intensity (Fig. 6F-I). H&E staining indicated that fewer tumor nodules were formed in metformin-treated group compared to untreated group. The number of tumor nodules was calculated (Fig. 6J, *P* = 0.0146). The lungs of *M.globosa*-infected mice treated with metformin displayed a decreased infiltration of CD206^+^ cells compared to *M.globosa*-infected mice (Fig. 6K, *P* = 0.0183). This finding suggests that *M. globosa* may contribute to lung cancer progression through immunometabolic remodeling of macrophages, and that metformin can counteract this effect in vitro and in vivo.

## Discussion

Despite growing recognition that intratumoral microbiota can drive cancer initiation and progression^23–25^, the role of the mycobiota in lung cancer progression is less understood. In this study, we analyzed the mycobiota composition in lung cancer patients across different clinical stages and identified higher abundance of *M. globosa* with higher clinical stages. Complementary in vitro and in vivo experiments further suggested intracellular *M.globosa* infection may promote lung cancer progression through OXPHOS-driven M2-like polarization of macrophages.

*M.globosa* was traditionally considered a skin commensal^26–28^. Indeed, recent studies have found the relationship between *M.globosa* and cancer progression. The amplicon-based sequencing has identified *M. globosa* in GBM, melanoma, lung cancer, breast cancer, and gastric cancer^7,16,29^. In breast cancer, *M. globosa* was found in the most aggressive tumors, correlating with worse prognosis^7^. In our study, the abundance of *M. globosa* correlates with patient outcomes, suggesting a promotive role of *M. globos*a in lung cancer.

Furthermore, in lung cancer patients, *M. globosa* was detected in both the alveolar lavage fluid (by metagenomic sequencing) and lung cancer tissues (by nested-PCR), and its presence has also been documented in human oral and nasal samples^30–32^. At present, how *M. globosa* reaches the lungs in patients remains unknown. One plausible route is inhalation through the oral or nasal cavity, followed by transport to the alveoli and eventually to lung tumor tissue. The precise mechanism of fungal entry into lung tumors merits further investigation.

Notably, the intratumoral fungi are mostly intracellular, and present in both cancer and immune cells^7^. Consistent with this, using 18S rRNA probe detection combined with macrophage marker staining, we observed colonized fungi in lung cancer tissues from patients and detected fungal signals within macrophages. However, it remains unknown whether these signals correspond specifically to *M.globosa*. Future species-specific validation will be necessary.

M2-like macrophage polarization is associated with a metabolic shift toward oxidative phosphorylation^20,33,34^. The intracellular infection of *M. globosa* markedly activated OXPHOS in macrophages, accompanied by oxoglutaric acid accumulation. It has been reported that oxoglutaric acid not only promotes OXPHOS metabolism but also reduces trimethylation of histone H3K27 in the nucleus, leading to upregulation of genes related to M2 polarization^35^. Both in vitro and in vivo, *M. globosa* infection drove macrophages toward an M2-like phenotype; in addition, PD-1 expression was elevated in vivo. We also infected macrophages with Malassezia pachydermatis, another Malassezia species detected in our patient cohort. Intriguingly, this fungus did not induce M2-like polarization or the associated metabolic alterations. These findings render a nonspecific fungal effect unlikely and suggest a species-specific role for *M. globosa* in promoting M2-like macrophage polarization.

M2-like macrophages can suppress anti-tumor immunity through multiple mechanisms, including secretion of IL-10 and TGF-β^36^ and expression of PD-1^37^. Consistently, conditioned medium from *M. globosa*-infected macrophages enhanced lung cancer cell proliferation and clonogenicity in vitro, and *M. globosa* treatment accelerated tumor growth in vivo.

Clinically, the abundance of *M. globosa* in ALF correlated with the proportion of CD163^+^PD-1^+^ macrophages in lung tumor tissues, and a higher proportion of these macrophages was significantly associated with poorer OS and PFS. These findings are supported by accumulating evidence that tumor-infiltrating PD-1-expressing macrophages correlate with poor clinical prognoses in cancer patients^38,39^. CD163 is widely recognized as a biomarker of M2 macrophages and its expression is associated with poorer clinical prognosis^40–42^; however, CD163 alone is insufficient to define macrophage polarization. Further studies with additional markers are required to confirm the M2-like polarization phenotype driven by *M. globosa* in human tissues. Together with previous reports, our findings suggest that intracellular *M. globosa* infection may contribute to remodeling the tumor immune microenvironment in lung cancer.

Metformin, an anti-diabetic drug, is currently being considered for cancer treatment^43–45^. Notably, metformin also inhibits mitochondrial Complex I activity, leading to suppression of OXPHOS^46^. We therefore examined whether metformin could counteract *M. globosa*-induced M2-like polarization of macrophages. Intriguingly, metformin reversed this polarization and consequently suppressed tumor growth, providing functional evidence for the involvement of metabolic reprogramming in our experimental models. Although its clinical application requires further mechanistic validation, the well-established safety profile of metformin positions it as a promising candidate for clinical translation.

Taken together, our findings add to emerging evidence that tumor-associated fungi are active components of the polymorphic microbiome in cancer^47^ and may contribute to lung cancer progression through immunometabolic remodeling of macrophages.

Several limitations of this study should be noted. Given the limited sample size of our clinical cohorts, additional multi-center data with larger sample sizes are needed to confirm the prognostic significance and generalizability. Our study found that intratumoral fungal abundance and immune cell infiltration correlate with patient outcomes. Further functional investigations are required to elucidate the underlying mechanisms. Moreover, the fungal burden administered to mice may not fully reflect the physiological levels in human lung tumor tissues or alveolar lavage fluid, although the fungal colonization in mice was performed using previously validated methods^46^. We acknowledge that the current data primarily demonstrate correlations. Although our metformin reversal experiments provide functional evidence supporting the involvement of metabolic reprogramming in M2-like polarization of *M.globosa*-infected macrophages, definitive proof will require future genetic loss-of-function studies. Finally, while current studies using experimental models suggest the involvement of *M. globosa* in lung cancer progression, the mechanism in human lung tumors remains to be elucidated.

Machine learning models may refine survival predictions by integrating clinical and biological variables^48–50^. Future studies integrating tumor mycobiome profiles, immune features, and clinical variables into machine-learning models may improve risk stratification and survival prediction in lung cancer.

## Methods

### Study population and sample selection

We enrolled 3 independent cohorts of NSCLC patients with different clinical stages according to the 9th edition of the AJCC TNM classification from the Second Xiangya Hospital between 2017 and 2022. Alveolar lavage fluid (ALF) and tumor tissues were collected during surgery and immediately stored at −80 °C. Patients with no prior antibiotic exposure and without clinical evidence of infection, sepsis, or active tuberculosis were enrolled. All patient treatments, sample collection, and study protocols were carried out following the guidelines of the National Health Commission of China and the ethical requirements outlined in the Declaration of Helsinki. Written informed consent to participate in the study was obtained from all patients. The study protocol involving human participants was approved by the Ethics Committee of the Second Xiangya Hospital, Central South University (Approval Number:LYEC2026-K0159).

For metagenomic sequencing, ALF was obtained from 28 NSCLC patients across different clinical stages, including 10 at stage 1 A and 18 at stages 1B–3. No adverse events were associated with the sampling procedure. ALF was collected under sterile conditions by instilling 20 mL of 0.9% NaCl into the alveoli and subsequently aspirating the fluid. In addition, surgically resected tumor tissues from these 28 patients were collected from the patients and immediately processed into liquid nitrogen or formalin-fixed paraffin-embedded (FFPE) blocks for immunofluorescence and 18S rRNA FISH. The clinical characteristics of the subjects are summarized in Supplementary Table 6.

Tumor tissues were collected from 56 NSCLC patients (28 at stage 1 A and 28 at stages 1B-3) to quantify the abundance of *M. globosa*The clinical characteristics of the subjects are summarized in Supplementary Table 7.

Tumor tissues and paired adjacent normal tissue samples were additionally collected from 18 patients,The clinical characteristics of the subjects are summarized in Supplementary Table 8.

### Metagenomic sequencing and analysis

The metagenomic sequencing was performed by the paired-end sequencing method on the Illumina platform (BGI, China). The genomic DNA was isolated from BAL and the whole DNA was sheared by ultrasonication (Covaris, Woburn, MA). The sheared DNA fragments were end-repaired (DNA End Repair Mix) at 20 °C for 30 min. The DNA fragments were purified by QIAquick PCR Purification Kit (Qiagen). Libraries were checked using Bioanalyzer 2100 (Agilent) and quantified using the ABI StepOnePlus Real-Time PCR System. Libraries were sequenced on an Illumina platform.

All the raw data were trimmed by SOAP nuke v.1.5.2. The trimmed reads were mapped to the host genome using SOAP2 software to identify and remove host originated reads. High-quality reads were de novo assembled using MEGAHIT software. Assembled contigs with length less than 200 bp were discarded in the following analysis. Genes were predicted over contigs by using MetaGeneMark. Redundant genes were removed using CD-HIT with identity and coverage cutoff 95% and 90%, respectively. In order to construct gene abundance matrix, Salmon software is used for quantification. After that, the read count table of species was rarefied to the minimum read counts to reduce the effects of uneven sampling in the cohort by using R package picante(Version 1.8.2)^51^. To estimate the microbial diversity, the observed species richness, Shannon-Wiener index, Gini-Simpson index and Pielou’s evenness and ACE estimator were calculated by R package vegan (10.1658/1100-9233(2003)014 [0927:VAPORF]2.0.CO;2). In addition, the R package mixOmics (version 6.10.9) was used to make partial least-squares discriminant analysis (PLS-DA) model. As for different analysis, the independent t-test was performed for each microbe by scipy package in python. The metagenomic sequencing data presented in this study can be found in NCBI Sequence Read Archive (SRA) database. The accession number(s) is SRA, PRJNA714488.

### Test for *M. globosa* enrichment

Tumor tissue samples were obtained from operating room as described above. The relative abundance of *M. globosa* was tested using nested PCR in newly diagnosed patients with NSCLC. Panfungal paired primers were FungiQuant yielding products of approximately 350 bp in the fungi 18S rRNA gene^52^. The specific primers of *M. globosa* were designed using Primer blast based on the 18S rRNA gene region, respectively for amplification. Meanwhile, all primers were validated using gradient qPCR to detect the annealing temperature and the specificity of primers. Primers were obtained from Sangon Biotech (Shanghai, China). The primers for *M. globosa* were MG-F: 5’- CAAATATGAAGGCGGGCTGGA-3’, MG-R: 5’-TGTCCTAGTGGTGGGCGAAC-3’ yielding products of approximately 100 bp.

The total DNA from tumor tissue samples including paired cancer tissue and adjacent cancer tissue were collected (Qiagen Blood & Tissue Kit, GER). The concentration of DNA was subsequently measured by Nanodrop. An equal volume of PBS was used as the negative control and the DNA from *M. globosa* strain was extracted as the positive control. The first round of amplification was performed in a 50 μl reaction mix containing 25 μl Premix Taq DNA polymerase (Takara, Dalian, China), 1.2uM for each primer and 5ul of template DNA. The temperature profile for amplification was as follows: initial denaturation at 94 °C for 4 min, denaturation at 94 °C for 30 sec, annealing at 55 °C for 30 sec, and extension at 72 °C for 1 min, for 35 cycles, followed by a final extension at 72 °C for 10 min. Two percent agarose gel electrophoresis was used (120 v 35 min) and the Panfungal DNA fragments were cut under the UV light and extracted by QIAquick Gel Extraction Kit (Qiagen, GER), whose concentration was detected by Nanodrop. An equal amount of DEPC treated water was also used to as a negative control. The reaction mixture (20 μl) for qPCR contained ChamQ Universal SYBR Color qPCR Master Mix (Vazyme Biotech, Jiangsu, China), forward and reverse primer (final concentration 400 nM), and the extracted Panfungal DNA fragments (5 ng). The Vazymecycling program was 40 cycles and consisted of 95 °C for 10 s and 56 °C for 30 s and 72 °C for 1 min with an initial cycle of 50 °C for 2 min and 95 °C for 2 min. Relative gene expression to control was calculated as follows:

$${\rm{Relativeabundace}}({\rm{i}})={((1/2)}^{{\rm{CTi}}})/(({1/2)}^{{\rm{CTc}}})={(1/2)}^{{\rm{CTi}}-{\rm{CTc}}}={(1/2)}^{\Delta {\rm{CT}}}$$

The cycle threshold values of the strain-specific primer (for strain i) and the universal primer (for total fungi) are denoted as CTi and CTc, respectively, with ΔCT representing the difference between these two values. According to the established equation, the logarithm of relative abundance exhibits a linear negative correlation with ΔCT^53^.

The primer sequences are shown in Supplementary Table 5.

### Culture of Cells and fungi

The cell line RAW264.7(Pricella, Cat. No. CL-0910) and murine Lewis Lung Cancer cells(Pricella, Cat. No. CL-0140) that were labeled with luciferase (Luci-LLC) were cultured in high-glucose DMEM medium containing 10% fetal bovine serum and passaged every 1–2 days as appropriate, free of fungal contamination. The cell line Thp-1(Pricella, Cat. No. CL-0233), A549(Pricella, Cat. No. CL-0016), PC9(Pricella, Cat. No. CL-0668) and H226(Pricella, Cat. No. CL-0396) were cultured in1640 medium containing 10% fetal bovine serum. *Malassezia globosa* (ATCC 96807) and *Malassezia pachydermatis* (BNCC357898) were cultured in 2693 Modified Dixon medium and Yeast Mold Medium (YM) liquid medium, respectively, with cultures kept free of miscellaneous infections.

### M.globosa infected macrophages

The RAW264.7 mouse macrophage line was cultured in high-glucose DMEM containing 10% fetal bovine serum (37 °C, 5% CO2). strains were cultured on mDixon at 30 °C for 1 week. *M.globosa* colonies were suspended in phosphate buffer and filtered through a strainer to obtain a spore suspension. An in vitro model of *M.globosa* infection of macrophages was established. Macrophages were counted by a cell counter at approximately 5 × 10^5^, inoculated in culture flasks and placed in an incubator for 12 hours. After the cells were adhered to the wall, *M.globosa* were added at different multiples of infection (MOI) at 37 °C. After the system was stabilized, the free *M.globosa* were washed away and the culture was continued for 24 or 48 hours.

THP-1 cells were treated with phorbol 12-myristate 13-acetate (PMA) at a concentration of 40 ng/mL for 24 h to induce macrophage differentiation (M0 phenotype). Following adherence, the macrophages were infected with *M. globosa* or *M.pachydermatis* at an MOI of 1 and incubated at 37 °C.

### The LLC-Luc mouse model

All animal experiments were performed in accordance with the guidelines of the Institutional Animal Care and Local Veterinary Office and Ethics Committee of the Hunan Normal University, China (License number: D2022058). Six-week-old C57BL/6 mice were purchased from Slake Experimental Animal Co., Ltd. (China, Shanghai). When injected with luci-LLC, mice were detected by in vivo imaging 3 weeks after the injection. All animal experiments were conducted in compliance with protocols approved by Central South University, China. We injected 1 × 10^6^LLC-luc cells into mice aged 8 to 10 weeks, and the clinical endpoint was reached when mice displayed signs of expiratory dyspnea. Anesthesia was administered using 2% isoflurane at 3 L/min fresh gas flow via a RWD multi-output animal anesthesia machine. Euthanasia was conducted by CO_2_ inhalation following the AVMA euthanasia guidelines.

### Pulmonary M.globosa transplantation experiment

To generate microbiota-depleted C57BL/6 mice, a regimen of intraperitoneal injections with broad-spectrum antibiotics cefoperazone (30 mg/kg) and an anti-fungal agent Fluconazole (2 mg/kg) was administered once daily for 5 consecutive days. Following the depletion of their microbiota, mice were nasally inoculated with 5 × 10^6^ CFU M*.globosa*^54^, while control mice received a PBS treatment. After seven days, both groups were intravenously injected with homozygous mouse Luci-LLC cells (1 × 10^6^) via tail vein. Luciferase activity was monitored on a weekly basis using non-invasive bioluminescence imaging conducted with the IVIS Lumina II (PerkinElmer). At week 6, lung samples were collected and subjected to H&E staining and PAS staining .

### Metformin treatment experiment

Microbiota-depleted C57BL/6 mice were produced by pretreatment with intraperitoneal injection of broad-spectrum antibiotics cefoperazone (30 mg/kg) and anti-fungal agent Fluconazole (2 mg/kg) one time per day for 5 days. Mice were infected with 5×10^6^ CFU *M.globosa* by nasal drip and controls were treated with PBS. Four mice per group were injected intravenously with 1×10^6^ homozygous mouse Luci-LLC cells 7 days later. Mice were treated with metformin (120 mg/kg) once every 3 days via intraperitoneal injection, while controls were treated with PBS. Meanwhile, luciferase intensity was monitored every week by non-invasive bioluminescence imaging using IVIS Lumina II (PerkinElmer). At week 6, lung samples were collected and subjected to H&E staining and PAS staining .

Metformin was added to the macrophage-fungi coculture systems at a final concentration of 100 µM for murine RAW264.7 cells and 120 µM for human THP-1-derived macrophages.

### Immunofluorescence

Paraffin sections were deparaffinized, rehydrated, and subjected to antigen retrieval. The sections were then incubated with fluorochrome-conjugated antibodies against CD206 (1:500, #24595, Cell Signaling Technology, Inc.), PD-1 (1:400, 18106-1-AP, Proteintech Group, Inc.), or CD163 (1:400, ab182422, Abcam PLC). After staining, the slides were mounted using Fluoromount-G and examined under a fluorescence microscope. Images were analyzed using ImageJ software.

### H&E staining

Tissue sections were stained with Harris’ hematoxylin for 6 h at 60–70 °C. Subsequently, they were rinsed in tap water until the water was clear. To differentiate the tissue, a solution of 10% acetic acid and 85% ethanol in water were used twice for 2 h and 10 h, followed by rinsing with tap water. Finally, the sections were soaked in a saturated lithium carbonate solution overnight, rinsed with tap water, and treated with eosin Yethanol solution for 2 days.

### Metabolomics analysis

Metabolomics analysis was performed using the Q300 Kit (Metabo-Profile, Shanghai, China). All targeted metabolites were quantified by UPLC-MS/MS (ACQUITY UPLC-Xevo TQ-S, Waters Corp., Milford, MA, USA). The optimized instrument settings were as follows: For HPLC, column: ACQUITY HPLC BEH C18 1.7×10-6 m VanGuard precolumn(2.1×5 mm) and ACQUITY HPLC BEH C18 1.7×10-6 m analytical column (2.1×100 mm), column temp:40°C, sample manager temp:10 °C; mobile phases: A: water with 0.1% formic acid and B: acetonitrile/IPA (70:30); gradient conditions: 0–1 min (5% B), 1–11 min (5–78% B), 11–13.5 min (78–95% B), 13.5–14 min (95–100% B), 14–16 min (100% B), 16–16.1 min (100–5% B), 16.1–18 min (5% B), flow rate: 0.40 mL/min, and injection vol.: 5.0 µL. For mass spectrometer, capillary: 1.5 (ESI + ), 2.0 (ESI-) Kv, source temp.: 150 °C, desolvation temp.: 550 °C, and desolvation gas flow: 1000 L/h. For data processing, the raw data files generated by UPLC-MS/MS were processed using TMBQ software (v1.0, Human Metabolomics Institute, Shenzhen, Guangdong, China) for peak integration, calibration, and quantification of each metabolite. The self-developed platform iMAP (v1.0, Metabo-Profile, Shanghai, China) was used for statistical analyses.

### Flow cytometry

Gating strategy of macrophages is shown in supplementary Fig. 5. Macrophages were harvested and washed with PBS. Dead cells were labeled by Zombie Aqua (BD). Macrophage cells from mice were stained by CD45-APCA-ly7, CD11B-PERP, PD-1-BV421, CD86-PE-ly7 and CD206-AF647. THP-1 derived macrophages were stained by CD45-FITC, CD86-BV650, CD163-APC. All the fluorescence -labeled antibodies were from BD Biosciences, CA. Cells were then analyzed using CytExpert software (Beckman Coulter, USA), and the results were further analyzed using FlowJo v10.0.7 software (BD Biosciences, CA).

### Cell viability analysis

The CCK-8 assay (K1018, APExBIO Technology LLC) was utilized to evaluate cell viability. A549, PC9, and H226 cells were seeded into 96-well plates at a density of 1000 cells/well. After a 3-hour incubation period with the CCK-8 reagent, the absorbance at 450 nm was measured using a Varioskan™ LUX microplate reader (Thermo Fisher Scientific, USA).

### Colony formation assay

For the colony formation assay, A549, PC9, and H226 cells were plated in 6-well plates at a density of 200 cells/well, with the medium being replaced every 48 hours. After 10 days of culture, the colonies were fixed and stained with crystal violet. Finally, the colonies were counted and analyzed.

### Measurement of Intracellular α-Ketoglutarate

Intracellular α-ketoglutarate (α-KG) levels in THP-1-derived macrophages were measured using the Amplex Red α-Ketoglutarate Assay Kit (S0323S, Beyotime Biotechnology, China), according to the manufacturer’s instructions.

### Fluorescent in situ hybridization (FISH) analysis

Following deparaffinization, FFPE sections were washed in 0.1 M Tris-HCl buffer (pH 7.4) for 15 min. Lysozyme treatment (10 mg/mL, 30 min) was applied to hydrolyze fungal cell wall components. Fungal burden in tumor tissues was then assessed via FISH using a specific 18S rRNA probe (5’-TTT AAG GGC CGA GGT CTC-3’, 2 μM, 50ul). This probe was conjugated to a Cy3 fluorophore (Ex/Em: 555/570 nm; Molecular Probes), and signals were captured using the Pannoramic 250/MIDI system.

### Statistical analysis

All data were analyzed using SPSS 20.0 and GraphPad prism 8. Continuous variables were analyzed using independent t-test or ANOVA, categorical variables using Fisher's exact test, and paired samples using paired t-test. Kaplan–Meier curves were used to assess PFS and OS. Univariable and multivariable Cox regression identified prognostic factors, presented as HRs with 95% CIs. Correlations were analyzed using Spearman’s rank correlation test. A significance level of *p* < 0.05 was considered statistically significant.

## Supplementary information

Supplementary information

## Data Availability

All the data generated or analyzed during this study are included in this published article. Additional data are made available in supplementary materials of this article. The metagenomic sequencing data presented in this study can be found in NCBI Sequence Read Archive (SRA) database. The accession number(s) is SRA, PRJNA714488 and can be accessible with the following link: https://www.ncbi.nlm.nih.gov/sra/PRJNA714488.

## Code availability

The underlying code for this study is available in NCBI Sequence Read Archive (SRA) database. The accession number(s) is SRA, PRJNA714488 and can be accessible with the following link: https://www.ncbi.nlm.nih.gov/sra/PRJNA714488.
