# Supplementary material for: M.globosa promotes lung cancer progression and M2 macrophage polarization through oxidative phosphorylation
Source: NPJ Precis Oncol. 2026 Jun 4;10:257. doi: 10.1038/s41698-026-01528-5 (PMC13332209; doi:10.1038/s41698-026-01528-5)
Supplement: Supplementary file 1 — Supplementary information [file 41698_2026_1528_MOESM1_ESM.pdf]

Supplemental information

M.globosa Promotes Lung Cancer Progression and M2 Macrophage Polarization through Oxidative Phosphorylation

Junqi Yi[1,3], Yiming Zhao[2], Zheng jiang Li[1,3], Anqi Chen[1,3], Ziying Tang[1,3], Leliang Zheng[2], Huabo Ge[1,3], Qian Yu[1.3], Wenliang Liu[1.3], Juanjuan Xiang[2]*, Jingqun Tang[1,3]*

1. Department of thoracic surgery, the Second Xiangya Hospital, Central South University, Changsha, Hunan, China
2. NHC Key Laboratory of Carcinogenesis and the Key Laboratory of Carcinogenesis and Cancer Invasion of the Chinese Ministry of Education, Cancer Research Institute, School of Basic Medical Science, Central South University, Changsha, Hunan, China
3. Hunan Key Laboratory of Early Diagnosis and Precise Treatment of Lung Cancer, Changsha, Hunan, China

*Corresponding authors: Jingqun Tang (tangjq@csu.edu.cn)
Juanjuan Xiang (xiangjj@csu.edu.cn)

Supplemental information

Inventory of Supplementary Information

Supplementary Data for Figures:

Supplementary Figure 1: The lung microbiota displays different composition between lung cancer patients with different clinical staging, related to Figure 1.

supplementary Figure 2: *M.globosa* is enriched in stage1b-3s with NSCLC, related to Figure 2.

Supplementary Figure 3: Intracellular infection of *M.globosa* induces oxidative phosphorylation of macrophages. , related to Figure 4.

Supplementary Figure 4: M2 polarization of macrophage induced by *M.globosa* is oxidative phosphorylation dependent, related to Figure 6.

Supplementary Figure 5: Gating strategy of macrophages.

Supplementary Table 1: The correlation between the abundance of 3 species in ALF and Clinical stages in NSCLC(n=28), related to Figure 1.

Supplementary Table 2: The correlation between *Malassezia globosa* abundance in ALF and clinical characteristics in NSCLC(n=28), related to Figure 1.

Supplementary Table 3: The correlation between *Malassezia globosa* abundance in tumor tissue and clinical characteristics in NSCLC(n=56),related to Figure 2.

Supplementary Table 4: Independent prognostic factors in NSCLC patients (Tumor, n=56), related to Figure 2.

Supplementary Table 5: List of all primers used in this study.

Supplementary Table 6: Clinical characteristics of patients(ALF n=28).

Supplementary Table 7: Clinical characteristics of patients(Tumor n=56).

Supplementary Table 8: Clinical characteristics of patients(Paired tissues n=18).

Supplemental Figures

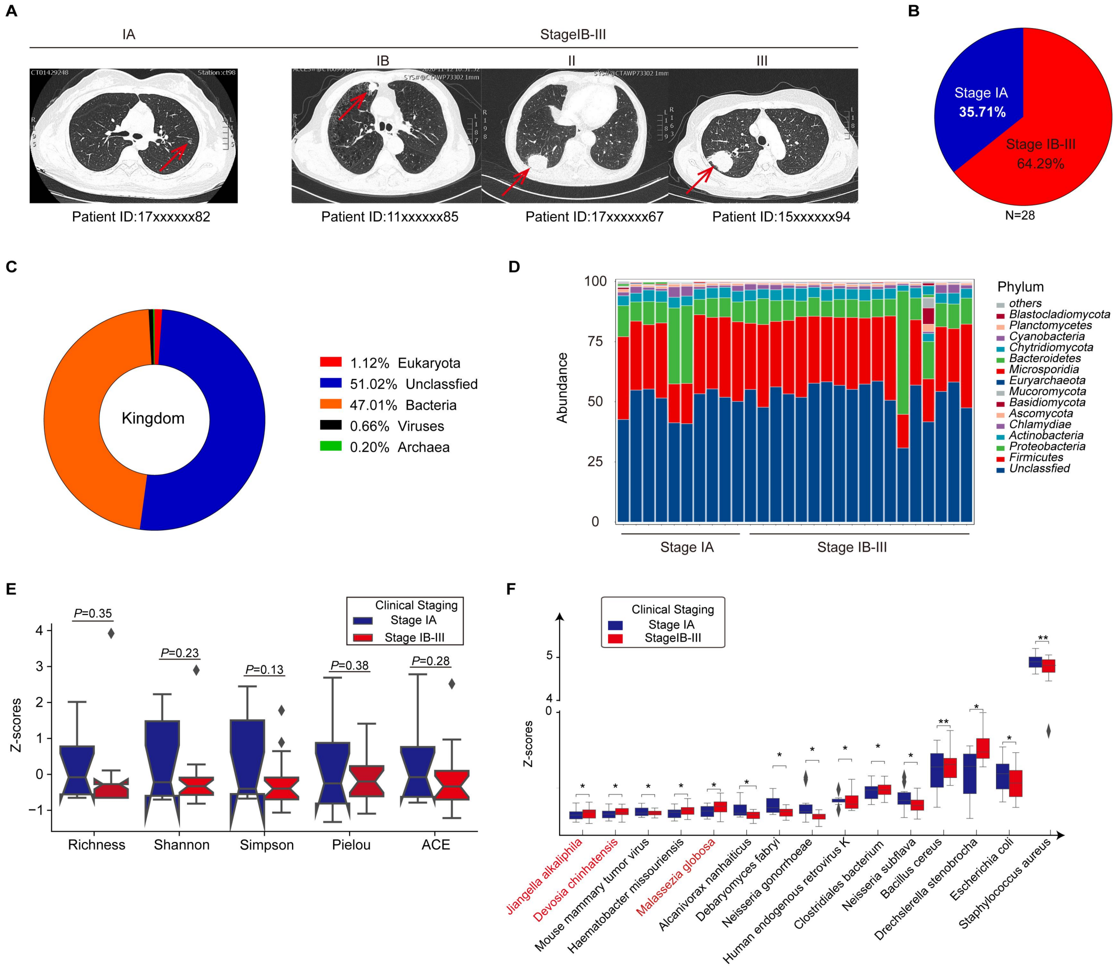

Supplementary Figure 1:The lung microbiota displays different composition between lung cancer patients with different clinical staging. (A)The 28 patients were divided into two groups according to Computed Tomography imaging; The red arrows indicated the tumor mass.(B)The pie chart shows percentage of stage 1A patients and stage 1B-3 patients.(C)The composition of microbiota in lungs; The kingdom taxonomic classification in lungs was shown;(D)Top 15 phyla in lungs.(E)The α-diversity including Richness index,Shannon index,Simpson index, Pielou index and ACE index in lung microbiome in NSCLC patients.(F)Top 15 species with significant differences between stage 1A and stage1B-3 of lung cancer patients. Species were identified by Wilcoxon rank-sum test.

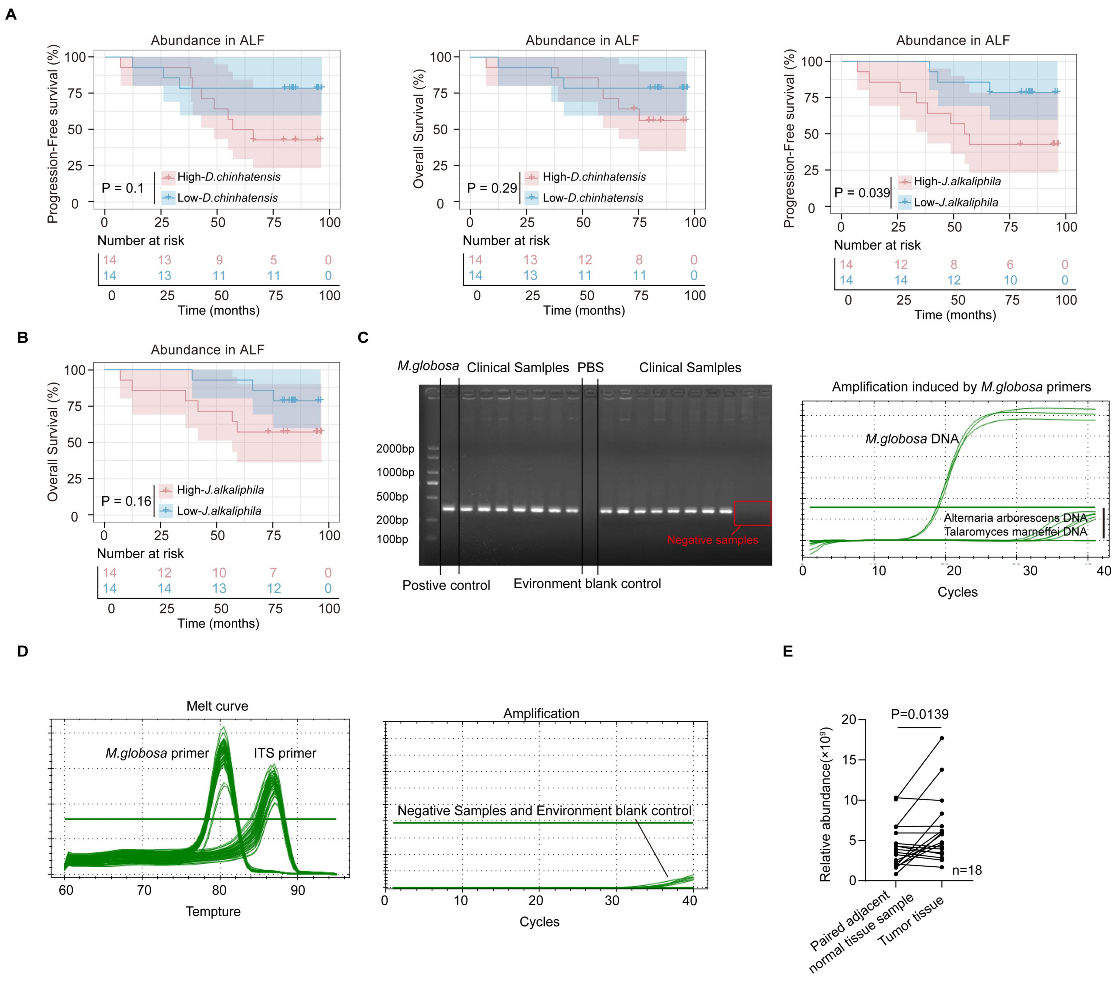

Supplementary Figure 2:*M.globosa* is enriched in stage1b-3s with NSCLC. (A and B) Progression-free and overall survival curves of NSCLC patients grouped into low or high based on median relative abundance of *D.chinhatensis* and *J.alkalipila* respectively, n=28. (C) Schematics of the nested-pcr experiments conducted in cancer and pan-cancer tissues of NSCLC patients to detect the relative abundance of *M.globosa*.(D) Left: Amplification curves of *M. globosa* DNA and other fungi DNA amplified with *M. globosa*-specific primers; Middle: Melting curves amplified with *M. globosa*-specific primers and ITS primers; Right: Amplification curves of PCR products from negative samples and environmental blank controls, generated by nested qPCR with *M. globosa* primers and ITS primers.(E).Assessment of the relative abundance of *M.globosa* relative abundance in paired tumor and adjacent normal tissues by nested PCR(n = 18).

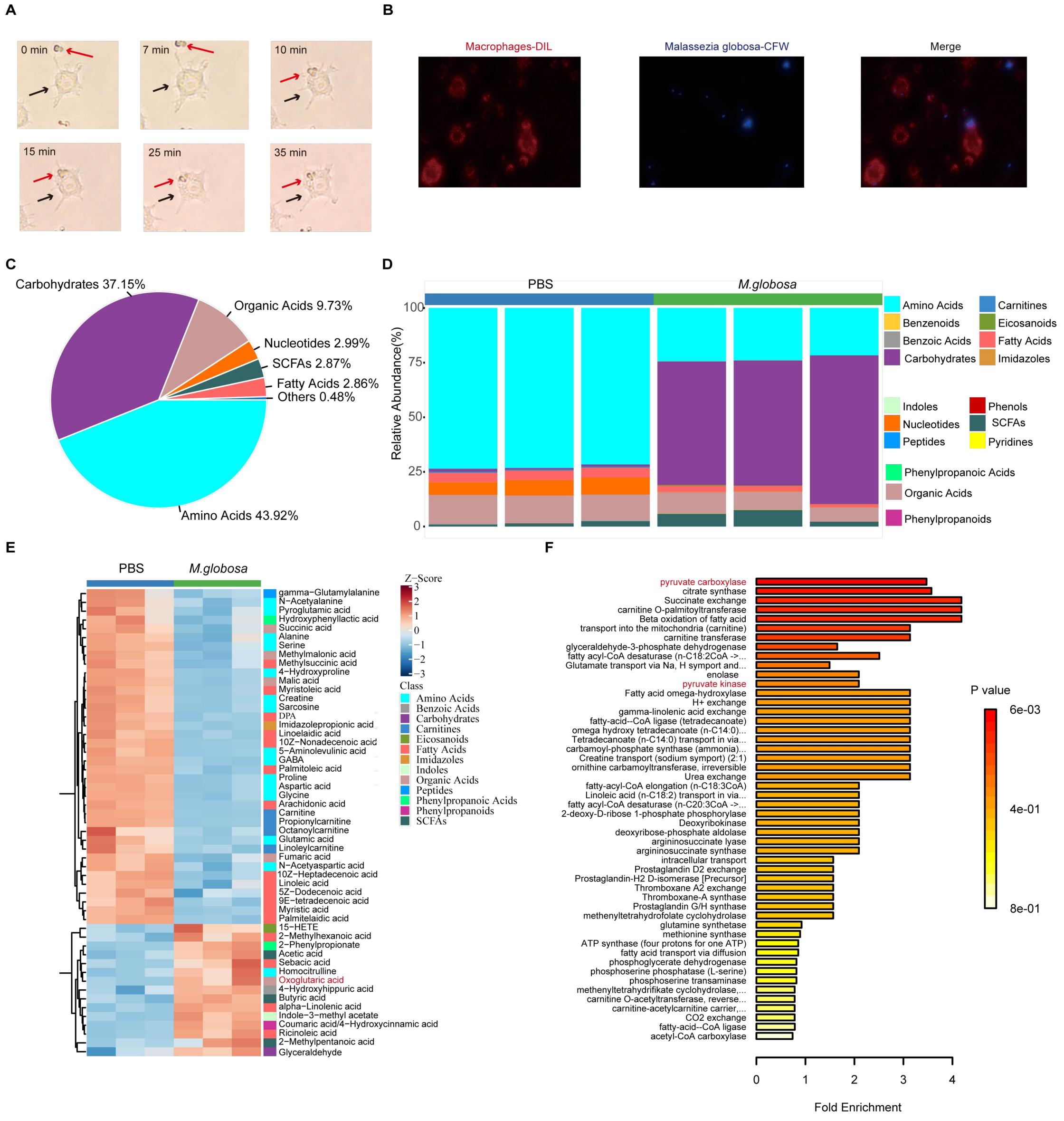

Supplementary Figure 3: Intracellular infection of *M.globosa* induces oxidative phosphorylation of macrophages. (A) The entry of *M.globosa* in macrophages was observed under microscopy. The red arrows indicated *M.globosa*. The black arrows indicated macrophages. (B)Intracellular infection of *M.globosa* into macrophages in fluorescent microscopy. Fungal cell wall of *M.globosa* was labeled with calcofluor white and cell membrane of macrophages was labeled with DID .(C)Global patterns of metabolites in RAW264.7. (D)Global patterns of metabolites in *M.globosa*-infected macrophages and uninfected control macrophages; (E)Differences in metabolites between *M.globosa*-infected macrophages and

uninfected control macrophages. (F)The highly enriched enzymes involved in cellular metabolism in *M.globosa* -infected macrophages.

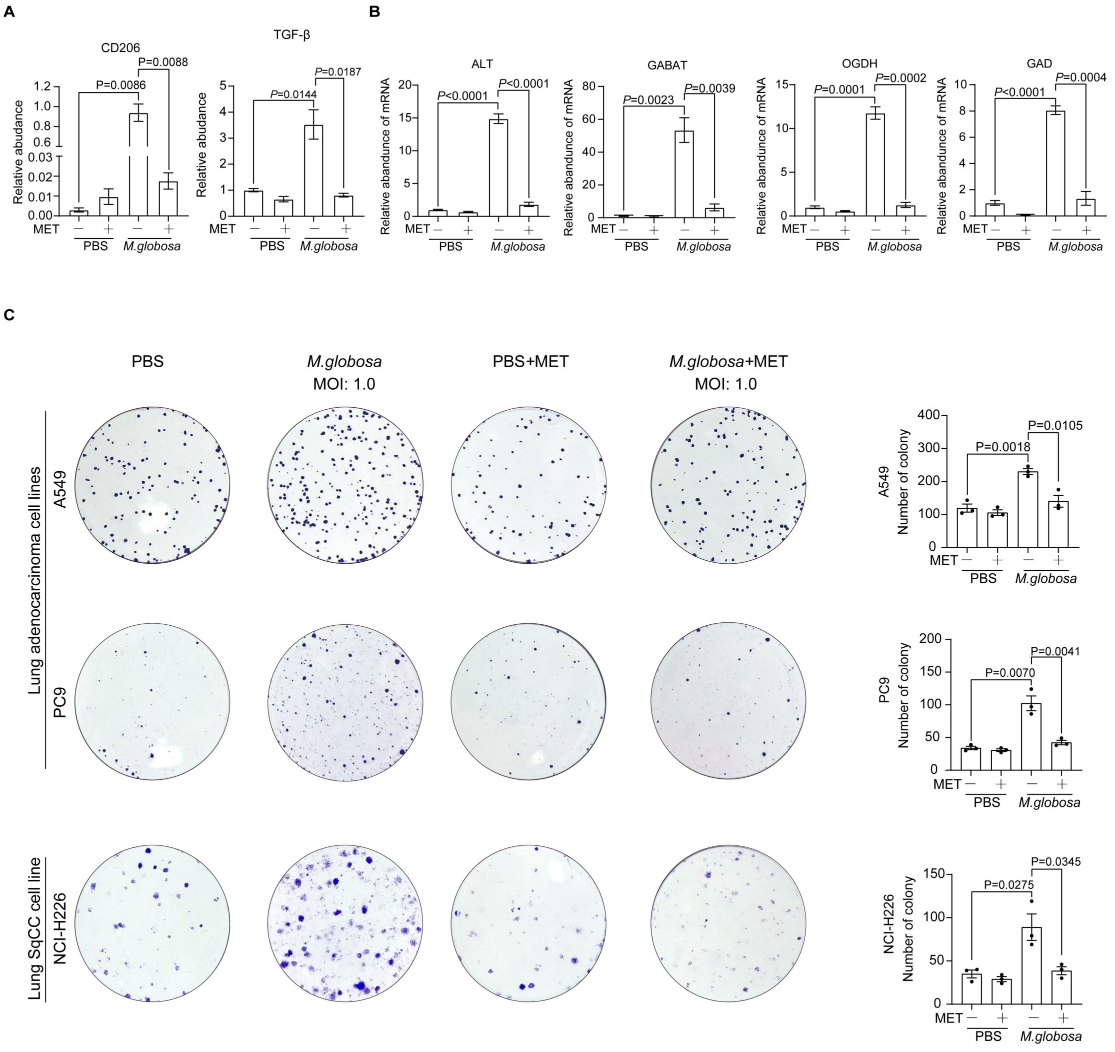

Supplementary Figure 4: M2 polarization of macrophage induced by *M.globosa* is oxidative phosphorylation dependent. (A)qPCR analysis of CD206, and TGF-β expression in RAW264.7 cells treated with metformin at 48h post-infection, n=3. (B).qPCR analysis of ODGH, GAD, GABA-T and ALT expression in RAW264.7 cells treated with metformin at 48h post-infection, n=3. (C) Colony formation assay showing the clonogenic potential of A549, PC9, and NCI-H226 cells treated with conditioned medium from metformin-treated, *M. globosa*-infected THP-1-derived macrophages (n=3).

A

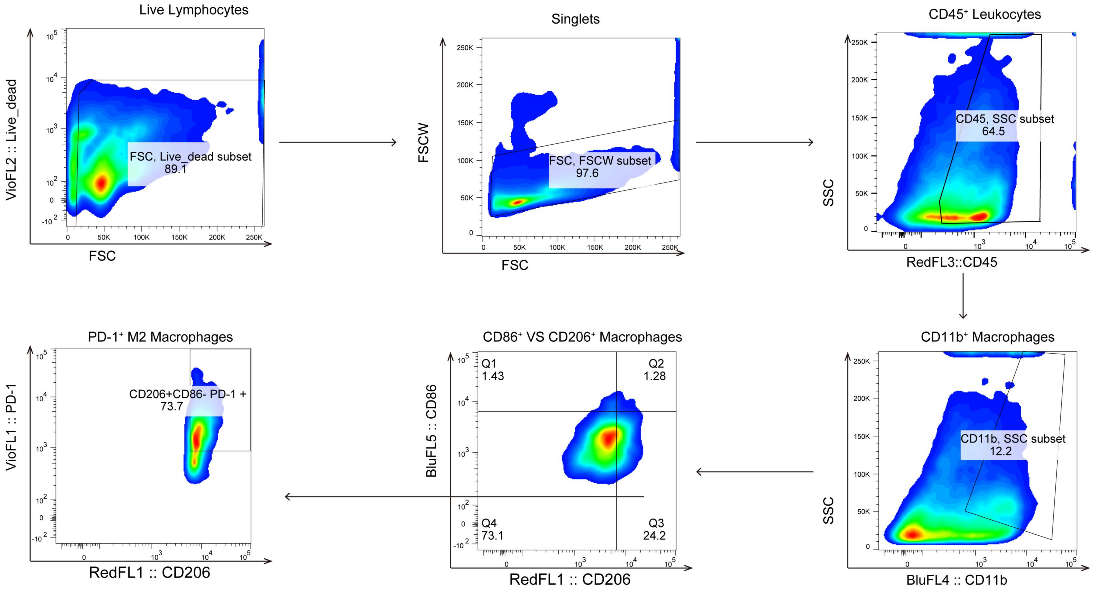

B

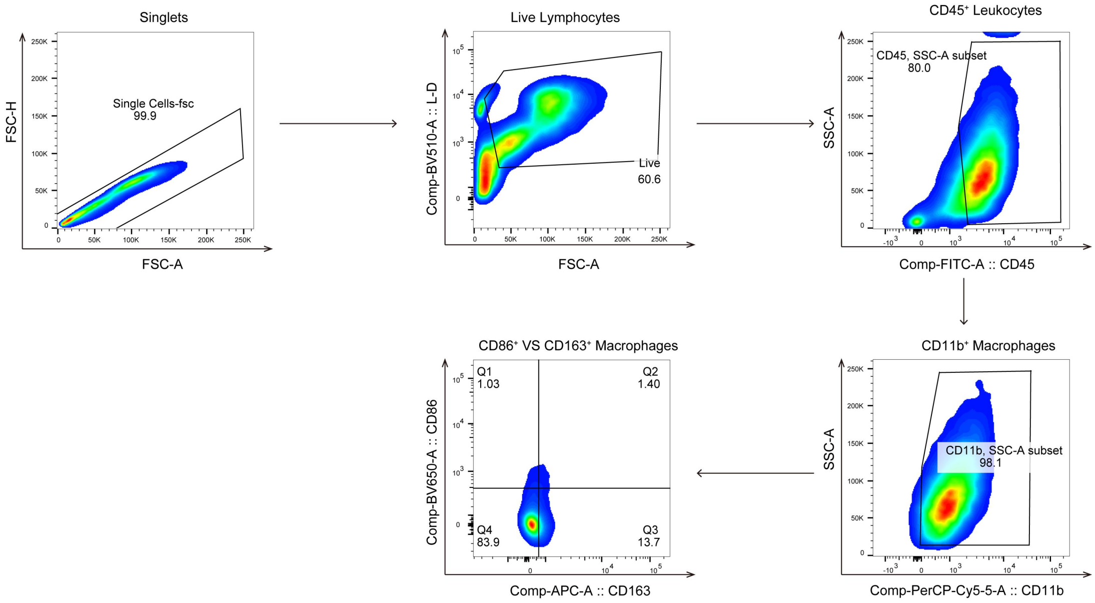

Supplementary Figure 5: Gating strategy for macrophages

(A) Gating strategy to identify macrophages in mouse cell populations.

(B) Gating strategy for THP-1-derived macrophages.

Supplementary Table 1. The correlation between the abundance of 3 species in ALF and Clinical stages in NSCLC(n=28)

Species	Case	Clinical stage I(n=17)	Clinical stage II-III(n=11)	P value
***M.globosa* abundance**				P value
Low	14(50%)	12(42.86%)	2(7.14%)	0.0183*
High	14(50%)	5(17.86%)	9(32.14%)	
***D.chinhatensis* abundance**				
Low	14(50%)	9(32.14%)	5(17.86%)	>0.9999
High	14(50%)	8(28.57%)	6(21.43%)	
***J.alkaliphila* abundance**				
Low	14(50%)	10(35.71%)	4(14.29%)	0.4401
High	14(50%)	7(25.00%)	7(25.00%)	
**Total**	28(100%)			

p value calculated using Fisher's exact test

Supplementary Table 2. The correlation between *Malassezia globosa* abundance in ALF and clinical characteristics in NSCLC(n=28)

Variable	Case	*M.globosa* abundance Low(n=14)	*M.globosa* abundance High(n=14)	P value
**Gender**				＞0.9999
Female	15(53.57%)	8(28.57%)	7(25.00%)	
Male	13(46.43%)	6(21.43%)	7(25.00%)	
**Age(years)**				0.7064
≤60	14(50.00%)	8(28.57%)	6(21.43%)	
＞60	14(50.00%)	6(21.43%)	8(28.57%)	
**Smoking history**				0.4197
Never	19(67.86%)	11(39.29%)	8(28.57%)	
Ever	9(32.14%)	3(10.71%)	6(21.43%)	
**Histological_type**				0.5956
Adenocarcinoma	24(85.71%)	13(46.43%)	11(39.29%)	
Squamous cell carcinoma	4(14.29%)	1(3.57%)	3(10.71%)	
**Total**	28(100%)			

p value calculated using Fisher’s exact test

Supplementary Table 3. The correlation between Malassezia globosa abundance in tumor tissue and clinical characteristics in NSCLC(n=56)

Variable	Case	*M.globosa* abundance Low(n=28)	*M.globosa* abundance High(n=28)	P value
**Gender**				0.3911
Female	38(67.86%)	17(30.36%)	21(37.50%)	
Male	18(32.14%)	11(19.64%)	7(12.50%)	
**Age(years)**				>0.9999
≤60	24(42.86%)	12(21.43%)	12(21.43%)	
＞60	32(57.14%)	16(28.57%)	16(28.57%)	
Smoking history				0.2823
Never	25(44.65%)	15(26.79%)	10(17.86%)	
Ever	31(55.35%)	13(23.21%)	18(32.14%)	
**Histological_type**				>0.9999
Adenocarcinoma	47(83.93%)	24(42.86%)	23(41.07%)	
Squamous cell carcinoma	9(16.07%)	4(7.14%)	5(8.93%)	
**Total**	56(100%)			

p value calculated using Fisher’s exact test

Supplementary Table 4: Independent prognostic factors in NSCLC patients（Tumor,n=56）

Variable	PFS						OS					
	Univariate analysis			Multivariate analysis			Univariate analysis			Multivariate analysis		
	HR	95% CI	P	HR	95% CI	P	HR	95% CI	P	HR	95% CI	P
**Gender**												
Male/Female	1.442	0.6725 to 2.905	0.3808				1.458	0.6823 to 2.921	0.3046			
**Age**												
≥60/＜60	1.143	0.4500 to 3.107	0.7824				0.9537	0.4965 to 1.866	0.8874			
**Smoking history**												
Ever/Never	0.985	0.5082 to 1.939	0.9644				1.195	0.4415 to 2.732	0.6971			
**Primary Tumor Size**												
＞3cm/≤3cm	4.547	1.758 to 13.10	**0.0026**	4.154	0.8142 to 75.76	0.1720	5.717	2.112 to 18.02	**0.0011**	4.254	0.8333 to 77.61	0.165
**TNM Stages**												
＞T1/T1	3.81	1.434 to 11.89	**0.0112**	1.321	0.1944 to 26.14	0.8051	4.961	1.752 to 17.64	**0.0052**	1.053	0.05174 to 7.868	0.9645
***M.globosa* abundance**												
High/Low	3.752	1.345 to 13.24	**0.0198**	2.490	0.7909 to 9.616	0.1444	3.556	1.257 to 12.63	**0.0267**	2.024	0.6376 to 7.916	0.2636
**Pathological type**												
Squamous cell carcinoma / Adenocarcinoma	1.205	0.4447 to 2.763	0.6836				1.195	0.4415 to 2.732	0.6971			

Supplementary Table 5: List of all primers and probe used in this study.

Name	Sequence (5′-3′)	Sequence (5′-3′)
**qPCR primers sequences for mouse**		
M-IL-10	F-AACTGCACCCACTTCCCAGT	R-CTCTTCACCTGCTCCACTGC
M-CD206	F-CTCTGTTCAGCTATTGGACGC	R-CGGAATTTCTGGGATTCAGCTTC
M-TGF-β	F-AGACCACATCAGCATTGAGTG	R-GGTGGCAACGAATGTAGCTGT
M-ALT	F-TCTGCACCTACCCAAACCTAC	R-GCACTGTAAGATCCCAAGCTG
M-GAD	F-CCACCAAGGTTCTGGATTTCC	R-GTACTTCAGGGTGTCTCTACAGT
M-GABAT	F-TGGGTTGCTTAGCGACCAC	R-GCTGGTTGTCCGTTGTAAACT
M-SSADH	F-AGAGGAAGCTCGCCGTATTTA	R-AGCAGGTTTCACCACCACAG
M-PDH	F-TGGTGCTGCTAATCAGGGTC	R-CCATAGCGGTTGTTCTCACAGA
M-CS	F-AGGCTAGACTGGTCACACAAT	R-AGGACAGGTAAGGGTCTGAAAG
M-IDH	F-ACAGGTGACAAGAGGTTTTGC	R-CTCCCACTGAATAGGTGCTTTG
M-OGDH	F-TATGGCCTACACGAGTCTGAC	R-CCAGCCGACGGATGATCTC
M-GAPDH	F-AGCTTCGGCACATATTTCATCTG	R-CGTTCACTCCCATGACAAACA
**qPCR primers sequences for human**		
H-CD163	F-TTTGTCAACTTGAGTCCCTTCAC	R-TCCCGCTACACTTGTTTTCAC
H-IL-10	F-GACTTTAAGGGTTACCTGGGTTG	R-TCACATGCGCCTTGATGTCTG
H-TGF-β	F-AACTGCTTCCTGTATGGGGTC	R-AAGGCGTCGTCAATGGACTC
**qPCR primer sequences for fungus**		
Malassezia_globosa	F-CAAATATGAAGGCGGGCTGGA	R-TGTCCTAGTGGTGGGCGAAC
ITS-FungiQuant	F-GGRAAACTCACCAGGTCCAG	R-GSWCTATCCCCAKCACGA
**Fish probe for fungus**		
18S rRNA probe	TTTAAGGGCCGAGGTCTC	

IL-10 **Interleukin-10,** ***CD206*** **mannose receptor, C type 1,** ***TGF-β*** **Transforming growth factor beta, CD163**

cluster of differentiation 163, *ALT* Alanine Aminotransferase, *GAD* Glutamate Decarboxylase, GABA-T Gamma-Aminobutyric Acid Transaminase, *SSADH* Succinic Semialdehyde Dehydrogenase, *PDH* Pyruvate Dehydrogenase, *CS* Citrate Synthase, *IDH* Isocitrate Dehydrogenase, *OGDH* Oxoglutarate Dehydrogenase. *M* mouse, *H* human, *ITS* Internal Transcribed Spacer. *18S rRNA* 18S Ribosomal RNA.

Supplementary Table 6. Clinical characteristics of patients(ALF n=28)

Clinical characteristics	Case
**Patients(n=28)**	
NSCLC patients	28(100%)
**Gender**	
Female	15(53.57%)
Male	13(46.43%)
**Age(years)**	
≤60	14(50.00%)
＞60	14(50.00%)
**Smoking history**	
Never	19(67.86%)
Ever	9(32.14%)
**TNM Stages**	
T1	15(53.57%)
T2-T4	13(46.43%)
**Clinical cancer Stages**	
IA	10(35.71%)
IB	7(25.00%)
II	8(28.57%)
III	3(10.71%)
**Primary tumor size(cm)**	
≤3	20(71.43%)
＞3	8(28.57%)
**Histological_type**	
Adenocarcinoma	24(85.71%)
Squamous cell carcinoma	4(14.29%)
**Total**	28(100%)

Supplementary Table 7 Clinical characteristics of patients(Tumor n=56)

Clinical characteristics	Case
**Patients(n=56)**	
NSCLC patients	56(100%)
**Gender**	
Female	38(67.86%)
Male	18(32.14%)
**Age(years)**	
≤60	24(42.86%)
＞60	32(57.14%)
**Smoking history**	
Never	25(44.65%)
Ever	31(55.35%)
**Clinical cancer stages**	
IA	28(50.00%)
IB-III	28(50.00%)
**TNM Stages**	
T1	31(55.35%)
T2-T4	25(44.65%)
**Primary tumor size(cm)**	
≤3	36(64.28%)
＞3	20(35.72%)
**Histological_type**	
Adenocarcinoma	47(83.93%)
Squamous cell carcinoma	9(16.07%)
**Total**	56(100%)

Supplementary Table 8. Clinical characteristics of patients(Paired tissues n=18)

Clinical characteristics	Case
**Patients(n=18)**	
NSCLC patients	18(100%)
**Gender**	
Female	9(50.00%)
Male	9(50.00%)
**Age(years)**	
≤60	10(55.56%)
＞60	8(44.44%)
**Smoking history**	
Never	8(44.44%)
Ever	10(55.56%)
**TNM Stages**	
T1	11(61.11%)
T2-T4	7(38.89%)
**Clinical cancer Stages**	
IA	9(50.00%)
IB-III	9(50.00%)
**Histological_type**	
Adenocarcinoma	15(85.71%)
Squamous cell carcinoma	3(14.29%)
**Total**	18(100%)
